# Decision aid prototype development for parents considering adenotonsillectomy for their children with sleep disordered breathing

**DOI:** 10.1186/s40463-016-0170-2

**Published:** 2016-11-04

**Authors:** Erin Maguire, Paul Hong, Krista Ritchie, Jeremy Meier, Karen Archibald, Jill Chorney

**Affiliations:** 1 Faculty of Medicine, Dalhousie University, Halifax, NS Canada; 2 IWK Health Centre, Halifax, NS Canada; 3 Division of Otolaryngology-Head and Neck Surgery, Halifax, NS Canada; 4 Faculty of Education, Mount Saint Vincent University, Halifax, NS Canada; 5 Division of Otolaryngology-Head and Neck Surgery, University of Utah School of Medicine, Salt Lake City, UT USA; 6 Department of Anesthesia, Pain Management and Perioperative Medicine, Halifax, NS Canada; 7 IWK Health Centre, 5850 University Ave, PO Box 9700, Halifax, NS B3K 6R8 Canada

**Keywords:** Decision aid, Shared decision-making, Obstructive sleep apnea, Pediatric otolaryngology, Informed consent

## Abstract

**Background:**

To describe the process involved in developing a decision aid prototype for parents considering adenotonsillectomy for their children with sleep disordered breathing.

**Methods:**

A paper-based decision aid prototype was developed using the framework proposed by the International Patient Decision Aids Standards Collaborative. The decision aid focused on two main treatment options: watchful waiting and adenotonsillectomy. Usability was assessed with parents of pediatric patients and providers with qualitative content analysis of semi-structured interviews, which included open-ended user feedback.

**Results:**

A steering committee composed of key stakeholders was assembled. A needs assessment was then performed, which confirmed the need for a decision support tool. A decision aid prototype was developed and modified based on semi-structured qualitative interviews and a scoping literature review. The prototype provided information on the condition, risk and benefits of treatments, and values clarification. The prototype underwent three cycles of accessibility, feasibility, and comprehensibility testing, incorporating feedback from all stakeholders to develop the final decision aid prototype.

**Conclusion:**

A standardized, iterative methodology was used to develop a decision aid prototype for parents considering adenotonsillectomy for their children with sleep disordered breathing. The decision aid prototype appeared feasible, acceptable and comprehensible, and may serve as an effective means of improving shared decision-making.

## Background

Patient- and family-centered care is recognized as an important part of pediatric medicine and shared decision-making between parents and providers is considered to be one of the pillars of family-centered care [1]. Shared decision-making involves using the best available evidence while incorporating patient values and preferences to make decisions that are supported by the patient, family and their healthcare providers [2]. Shared decision-making is reported to result in a number of benefits including improved patient and family experience, improved treatment adherence and disease control, and promotion of more effective use of health care resources [1, 3]. Furthermore, not engaging in shared decision-making can lead to decisional conflict which is defined as the uncertainty as to what choice should be made [4]. Decisional conflict may be caused by lack of information, unclear patient or family values, emotional distress, and perceived pressure from others, all of which may be mitigated by shared decision-making. Although there is variation in the preferred level of involvement in medical decision-making across patients and family members and contexts [5, 6], perceiving less decisional control than desired is associated with higher levels of decisional conflict [7].

Using a *decision aid* to assist in medical decision-making has been shown to decrease the proportion of patients who are passive members in the decision-making process, increase the number of patients with realistic ideas about the risks and benefits of a medical procedure, and reduce decisional conflict [5]. Decision aids can empower the patient with information so that they may have a more meaningful and informed discussion with their physician [8]. Typically, a decision aid includes a description of the health condition and management options being considered, a summary of the evidence for each of these options including risks and benefits, and an element to help the patient consider this information in the context of their personal values [9].

Research suggests that parents want to be involved in healthcare decision-making for their children [10, 11]. However, studies in pediatrics to date have demonstrated that shared decision-making may be limited in clinical practice [12], and the format in which information is provided to parents currently does not facilitate adequate recall of information shared during consultations [12–14].

In pediatric otolaryngology, surgical procedures such as adenotonsillectomy have many benefits but also involve inherent risks [15, 16]. It is often unclear whether these procedures are always necessary considering that many children will outgrow the problems these surgeries are deigned to treat [17]. Due to the limited consultation time with surgeons, complexity of information on the risks and benefits of these procedures, potential of multiple procedures being considered at once (e.g., tonsillectomy and tympanostomy tube insertion), and the need to often consider the perspective of more than one caregiver [10], evidence-based decision support tools have potential utility in pediatric otolaryngology settings.

There has been a recent call for the development of decision aids in otolaryngology [9], but to date no comprehensive evidence-based decision aids are available. Thus, the objective of this study was to describe the development process of a decision aid prototype for parents considering adenotonsillectomy for their children with sleep disordered breathing.

## Methods

Local Institutional Review Board approval was obtained (IWK Health Centre). This study was part of a larger mixed-methods research project examining shared decision-making in pediatric otolaryngology. The data on decision outcomes from the needs assessment sample have been previously reported [18].

### Overview of study design

**Fig. 1:**
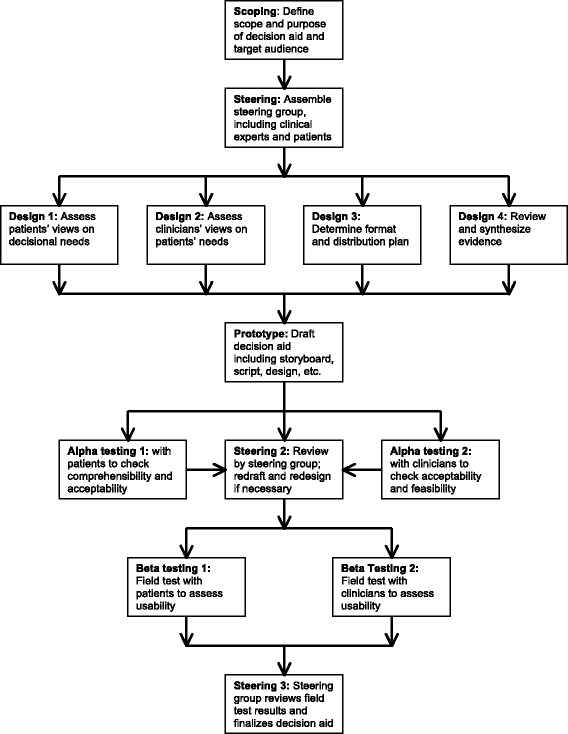
The International Patient Decision Aids Standards (IPDAS) framework

The current decision aid prototype was developed using the process defined by the International Patient Decision Aids Standards (IPDAS) Collaboration (Fig. 1) [19]. The IPDAS outline a framework to guide the creation of decision aids and highlights the importance of multi-informant, multi-method assessments of decision needs, using the most up-to-date information, including critically appraised and comprehensive evidence, gathering feedback from patients and providers, and testing the decision aid in real-world settings [20]. This manuscript outlines the initial phases of decision aid prototype development including 1) establishing a steering committee, 2) conducting a needs assessment, 3) designing the decision aid prototype, and 4) assessing the comprehensibility, feasibility and acceptability of the decision aid prototype, and modifying it accordingly.

1. Steering committee
  Participants in the steering committee included one fellowship trained pediatric otolaryngologist working in an academic tertiary care pediatric health centre with expertise in patient-centered care research, one child health psychologist with expertise in perioperative care, one cognitive psychologist with expertise in shared decision-making, two parents of children who previously had elective pediatric otolaryngology procedures, one nurse with expertise in patient-provider communication and shared decision-making, and one nurse research coordinator with expertise on perioperative care research.
2. Needs assessment
  Participants in the needs assessment phase included 41 parents of children involved in the larger mixed-methods study (see Gorodzinsky et al. [18] for demographics on the needs assessment sub-sample). Semi-structured interviews were conducted by telephone calls with parents two weeks after their child’s surgical consultation. Interviews queried perceived need for decision support (including barriers and facilitators of decision-making), information perceived as important in decision-making, and preference for format of delivery of decision support. Qualitative thematic content analysis was used to summarize the results [18]. Videotapes of the surgical consultations with participants included in the larger study were also reviewed to identify the most common risks and benefits discussed.
3. Prototype development
  The steering committee members reviewed the results of the needs assessment and drafted the initial prototype version of the decision aid. A description of obstructive sleep apnea and treatment options was documented by the nurse research coordinator and refined by the otolaryngologist. The risks and benefits included in the decision aid were those that were identified as important by parents and those that were most commonly discussed during the videotaped consultation visits.
  To further populate the risks and benefits section of the decision aid and to add details of the risks and benefits (e.g., percentage of bleeding post-surgery), a scoping literature review of guidelines and reviews of the risks and benefits of adenotonsillectomy for obstructive sleep apnea was conducted (see Table 1 for search strategies). Databases searched included PubMed, Embase, Cochrane, and National Guidelines Clearinghouse; hand searching of relevant bibliographies were also conducted. Inclusion criteria for the scoping review were: participants under 18 years of age, published in English, and reporting was in the format of a Cochrane review, systematic review or clinical guideline endorsed by recognized medical societies (e.g., American Academy of Otolaryngology-Head and Neck Surgery). A pediatric otolaryngologist and research assistant screened the titles and abstracts (163 papers identified in the initial search) and 38 articles moved forward at the abstract review level. Another research assistant reviewed the full text of these articles and 16 papers were excluded based on the inclusion and exclusion criteria. Two additional papers that were excluded at the abstract level were added back into the review (originally excluded because they contained overlapping content with conditions other than obstructive sleep apnea); thus, a total of 24 articles were included in the final review.

**Table 1:** Search strategy of the risks and benefits of adenotonsillectomy for obstructive sleep apnea

Database	Search terms^a^	Number of results
PubMed	Search obstructive sleep apnea Filters: Guideline; Systematic Reviews; Meta-Analysis; Child: birth-18 years	117
Embase	‘obstructive sleep apnea’/exp OR ‘obstructive sleep apnea’ AND ([adolescent]/lim OR [child]/lim) AND ([cochrane review]/lim OR [systematic review]/lim OR [meta analysis]/lim)	46
Cochrane library	“obstructive sleep apnea” AND “children” AND “treatment”	7 (Cochrane articles) 4 (Reviews)
National guidelines clearinghouse	“obstructive sleep apnea” AND “children” AND “treatment”	25

^a^Literature search done on July 18, 2014
  The steering committee decided to start with a paper-based format of the decision aid with the aim of developing a complementary electronic platform in the future. This was done to minimize costs and to gather more information about the acceptability and comprehensibility of the decision aid prototype.
4. Comprehensibility, acceptability, and feasibility testing
  Participants in this phase included three additional otolaryngologists (two from the primary centre and one from another academic centre), four otolaryngology clinic nurses, and four parents of children who had previously undergone adenotonsillectomies. Participants in this phase were not involved in the initial development of the decision aid prototype.

To conduct the comprehensibility, acceptability and feasibility testing, we used a combination of think aloud techniques and semi-structured interviews [21]. In the think aloud technique, participants were asked to comment on the decision aid as they navigated it, suggesting areas for improvement. Participants were provided with a pen to note their changes directly on the prototype. The semi-structured interviews queried the comprehensibility (degree to which the content of the decision aid was understandable), acceptability (degree to which the decision aid added value to the consultation), feasibility (degree to which the decision aid would fit into practice), and desirability (degree to which the decision aid was presented in a visually appealing way). Participants were provided with the criteria for each construct (e.g., for comprehensibility: “the tool is easy to understand”) and asked to comment on whether the decision aid met the criterion or required minor or major revisions to meet the criterion (Table 2). If revisions were indicated, the participants provided details on their suggested changes. Interviews were audio-recorded, transcribed verbatim and checked for accuracy. Qualitative content analysis was used to analyze and report the interview data [18]. Think aloud and interview results were analyzed together as a complete dataset.

**Table 2:** Decision aid prototype feedback semi-structured interview guides

Interview guide	
Comprehensibility	Do you think the tool is easy to understand in this format? Do you think the tool will be easy to use in this format? Would you have liked to have this tool presented to you by your child’s surgeon to use during a consultation? (Parents only) Would you have used this tool during the surgical consultation if it had of been available to you? (Parents only)
Acceptability	Do you think using the tool will help parents feel more involved in the decision making process? Do you feel the tool will be useful for parents to use with their surgeon during the consultation? Do you think surgeons using the tool with parents will help them in the process of making a treatment decision? Do you think the tool will help surgeons better understand the patients and parents values and concerns about their treatment decision? Do you feel that this tool clarified questions or concerns parents may have? (Parents only)
Feasibility	Do you feel the tool will be of benefit in the consultation appointment? Do you think use of the tool during a consultation will have an influence on the clinic process? Do you think this tool would fit into your practice? (Surgeons only) Do you think you would use this tool in your clinical practice? (Surgeons only) Do you think other hospitals should provide this tool to parents during an ENT consultation? Would you have liked to have this tool to take home and refer to after your consultation with your child’s surgeon? (Parent only)
Desirability	Do you think the tool is visually appealing? Do you think the tool has a polished presentation? Is there anything that you would like to change about the tool? Is there anything that you specifically like about the tool? Do you have any further comments you would like to add?

Testing was conducted in iterative cycles. The first cycle involved six participants; the second involved five participants; and the third involved three participants. Analyses were conducted between each cycle and the decision aid prototype was refined based on these data after each cycle.

## Results

1. Steering committee
  The steering committee met nine times throughout the project as a group and subsets of the steering committee met an additional 15 times to review results and plan the next steps.
2. Needs assessment
  Previous studies conducted by our group demonstrated that many parents experienced clinically significant decisional conflict when deciding whether their child should undergo elective pediatric otolaryngology surgeries [11, 22]. In a pilot study looking at shared decision-making in pediatric otolaryngology, parents’ perceptions of shared decision-making were negatively correlated with decisional conflict [11]. However, analysis of videotaped data (e.g., amount of talking done by parent, number of questions asked) did not correlate with perceptions of shared decision-making, suggesting there may be a discrepancy in how parents’ perceive their involvement versus their actual engagement during the surgical consultation [23]. Overall, the conclusion was that many parents experienced decisional conflict and strategies to increase shared-decision making (e.g., via decision support tools) are needed for parents considering elective pediatric otolaryngology surgeries.
  Qualitative interviews showed that parents wanted a decision support tool [18]. Those parents interviewed noted their main reason for referral was for further investigation of their child’s symptoms and to discuss possible treatment options. Many parents interviewed were accessing the Internet and relying on their own personal surgical experience as a guide for additional information as they sought to make a treatment decision for their child. The use of electronic means to gain this information was reported to be important for families so that they can readily access the information at a convenient time. Parents also noted that they would prefer to have access to a reliable vetted source of information directly pertaining to their child’s condition and possible treatment options. Specifically, parents wanted a tool developed by a reputable source (e.g., their healthcare facility) that contained information on the procedure itself, the risks and benefits of the procedure, as well as symptoms that their child is experiencing. Of significance was that parents themselves had differing opinions as to the optimal timing of delivery of a decision support tool that would be most beneficial for them. That is, some parents thought a decision aid would be the most helpful before the consultation to help them prepare for the visit and to ensure a well-thought out treatment discussion, while some parents wanted to talk to their surgeon first before receiving the decision aid to reduce unnecessary stress.
3. Prototype Development
  The decision aid prototype included the following three main sections: 1) a brief description of pediatric obstructive sleep apnea and the main management options, 2) information on these options including risks and benefits, and 3) a section on values clarification (Fig. 2). As recommended by the IPDAS Collaboration, the decision aid was written using language not greater than eighth-grade level. Although several parents reported that they would like an electronic version of a decision aid [18], we began with a cost efficient paper format to gather initial impressions on the design and content before proceeding to an electronic version.

**Fig. 2:**
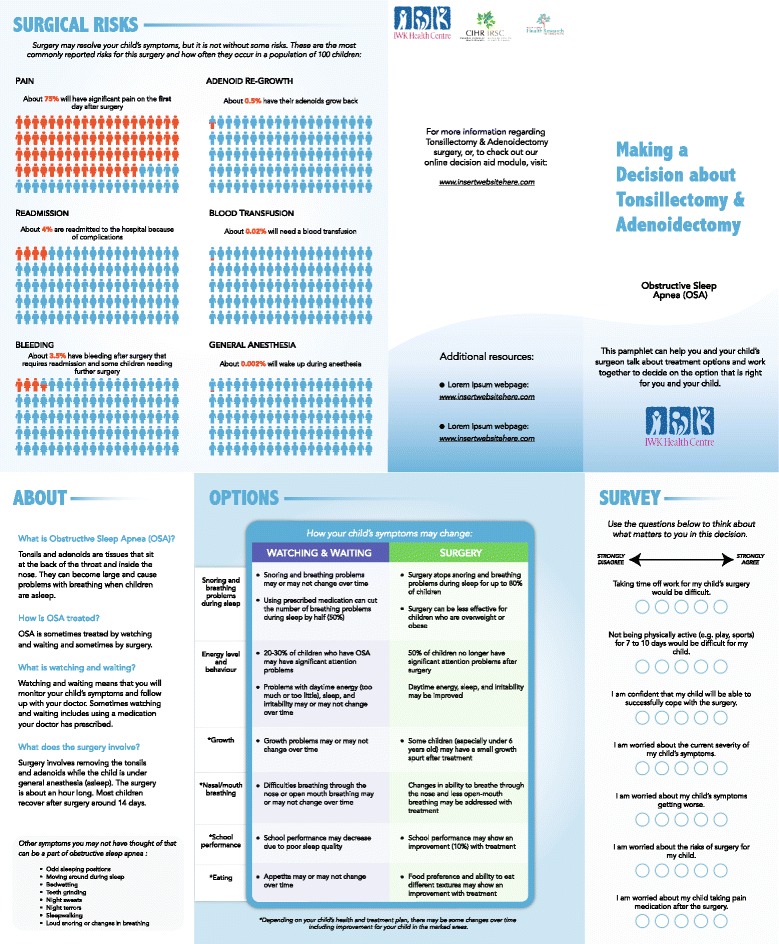
The decision aid prototype
  The prototype included a matrix that allowed easy comparison between the two primary courses of treatment (watching and waiting versus surgery). A matrix or options grid has been suggested to be a helpful way to display this information in an organized fashion to permit straightforward comparison of treatment options [9]. Content areas in the matrix included effects of each treatment option on: snoring and breathing problems during sleep, energy and behaviour, school performance, nasal/mouth breathing, growth and eating. A separate section outlining the surgical risks including pain, bleeding, general anesthesia, readmission, adenoid re-growth and the possible need for blood transfusion was included. To improve comprehensibility, occurrence rates of each risk were described in percentages and shown visually using pictographs [9].
  The decision aid prototype also included an exercise for values clarification. Values included: worry about risks, ability to take time off from work for parents, physical inactivity for the child for a period of time, confidence in child’s ability to cope with surgery, worry about symptoms getting worse, and parents concern of their child taking pain medication after surgery (themes derived from needs assessment phase). The prototype provides a bubble scale that allows parents to consider each value and indicate the degree to which they agree or disagree with each statement. This rating system provides parents with a visual representation of their thoughts related to the condition and procedure, and aims to provide them with some clarification of their values on this topic.
4. Comprehensibility, acceptability, and feasibility testing
  Multiple iterative cycles were used to test comprehensibility, acceptability, and feasibility of the decision aid prototype.

**Cycle 1.** Cycle 1 of evaluation included six participants (two parents, two nurses, two fellowship-trained pediatric otolaryngologists, Table 3). Three participants indicated that the prototype met criteria for comprehensibility and three suggested minor edits. The primary theme for comprehensibility edits was wording changes. Suggestions were made for wording of recommendations (e.g., de-emphasizing nasal spray in the watching and waiting option), clarification of surgical risks (e.g., editing wording for general anesthesia), and revision to the watchful waiting definition (e.g., the parent could be the one who is monitoring their child and following up with the doctor as necessary).

**Table 3:** Feedback from Providers and Parents (Cycle 1)

Domain	Changes suggested	n	Sample quotes/Edits
Comprehensibility	None	3	“Yeah, I think it’s a good general format because I think over time, what we all do and depending on the busyness of the day, it’s a great tool to bring all the surgeons back to the same key points for perspective.” “So when you kind of have something laid out like this, I think that would be helpful for families to just make them aware if they can give this a little bit more time or not, and to really see on paper, sometimes it’s really helpful too…” “Considers other factors” many families get tunnel vision in an appointment and then post op are wishing they had considered other factors.”
	Minor	3	“I think the doctor is not really keeping an eye on the symptoms. You will keep an eye on the symptoms and revisit the doctor as necessary or possibly revisit the doctor if things are the same or worsening.” Concern with the language used in the risks general anesthetic section will be of concern to families especially when they take it home. “…when I do the teaching, from a child’s perspective, it is really scary…” Provider added in pain management to the values section. “there are a certain number of kids that won’t take pain medicine, so that’s a worry for parents sometimes.”
Acceptability	None	6	Using the tool to help parents feel more involved “I think it will, for those who want to be involved.” “…I think that it can only serve to benefit the relationships between us, the client or the parent and the medical doctor if they’re able to understand the stress that the parents are going through…” “… it may be the 5^th^ or 10^th^ surgery for that Dr. in that week, hypothetically, but it’s the biggest decision that I would ever make because the boys are everything…” “My biggest like is that it makes parents recognize that not everyone needs surgery… the easiest thing to do is something, sometimes the hardest thing to do is nothing.”
Feasibility	None	6	“And I would feel confident feeling that I was getting something from the hospital… that I could just kind of read with confidence knowing…” “… I’d like to be able to share it with my mom and my family, just my support system in trying to help lead me through to get to where we needed to get to with regards to putting our children through a surgery.” Will have a negative impact on clinic flow initially, but “I think at surgery and after surgery parents will be better informed and may have less questions/concerns.” “Efficiency has to be important, so I don’t think I know where the right place to apply this…” Would need to pilot first
Desirability	None	4	“… the values really bring in the human part of it, what it means… other real life issues that are impacting somebody’s ability.”
	Minor	2	Concern with the 3 back panels’ parents would miss a page; perhaps consider placing a “last” page as a resource section with links. “…the general anesthetic risk because when I do the teaching, from a child’s perspective, it is really scary to think that they might wake up…just having it there, I think it’s terrifying.”

One participant recommended adding a value around pain medication to the values clarification section. All participants indicated that the tool was acceptable and added value to the consultation; no edits were suggested. For acceptability, participants generally indicated that the decision aid would increase consistency and provide a source of reliable information for parents. In terms of feasibility, although all participants indicated that no edits were needed, the main theme in comments (particularly from providers) was that the decision aid could slow down consultation times. One participant highlighted the importance of piloting the decision aid. In terms of desirability of design, participants suggested minor changes in the layout and an edit for how surgical risks are presented (suggested presenting the percentage of patients who do not have complications to show outcomes more positively).

On the basis of Cycle 1 results, the decision aid was edited for wording, pain was added as a value, and presentation of risks were modified (e.g., adding a timeline for pain, wording of risks for anesthesia changed from “problems waking up” to “significant complications”). We did not change how the risks were presented as suggested by one participant because no other participants voiced any concerns and since previous decision aids developed using the IPDAS framework have used a similar format (i.e., presenting percentage of risks).

**Cycle 2.** Cycle 2 included five participants (two parents, two nurses, one fellowship-trained pediatric otolaryngologist, Table 4). All participants in Cycle 2 were different than those from Cycle 1. Four participants indicated that the decision aid was comprehensible in its current format. One participant suggested minor edits for comprehensibility (highlighting a value related to symptom intensity). Four participants also indicated that the decision aid was acceptable and added value to the consultation. One participant suggested adding sections on postoperative recovery. Feasibility was rated as acceptable by all participants. Desirability was rated as acceptable by four participants. One participant suggested minor edits to the color scheme.

**Table 4:** Feedback from Providers and Parents (Cycle 2)

Domain	Changes suggested	n	Sample quotes/Edits
Comprehensibility	None	4	
	Minor	1	“Could there be a question asking: I am worried that these symptoms are impacting my child’s overall health or quality of life… it might be helpful to understand how severe the parents feel the problem is currently…”
Acceptability	None	4	“I definitely would have wanted it at consult especially the values.”
	Minor	1	“The only thing that I don’t see on here … about fever after the fact because that was the one thing that I had to call back about … maybe there should be a section to show post- surgery what to expect.”
Feasibility	None	5	Will have a positive flow “give the family a sense of protocol, visually gives the impression of both options in specific circumstances”. “This aid may help make the informed consent process more complete…for others with significant decision conflict this may make the encounter more efficient.” “…may increase the length of visit for some patients- particularly if there is NO decision conflict and the parents already have their mind made up regarding a decision prior to the consultation…”
Desirability	None	4	“I like the images showing the percentages for specific risks, making this appear more granular for the parents. It accurately and succinctly gives the pros and cons of surgery versus watchful waiting.”
	Minor	1	“I probably would have used something brighter … even have two different colors for waiting and surgery, so they don’t look like you’re reading across the same information”.

On the basis of results from Cycle 2, an additional value of perceived severity of symptoms was added to the decision aid. Given the scope of the decision aid, we did not expand to include postoperative recovery course. We postponed changes to the color scheme until after feedback from design experts in Cycle 3.

**Cycle 3.** Cycle 3 included review by three design professionals (one medical illustrator/Internet designer and two design experts, Table 5). These participants rated the decision aid as comprehensible, acceptable and feasible, but all had comments about the desirability of the decision aid. Not surprisingly, the theme for these comments was formatting and design (e.g., color choice, alignment, presentation).

**Table 5:** Feedback from Design Professionals (Cycle 3)

Domain	Changes suggested	N (%)	Sample quotes/edits
Comprehensibility	None	3	
Acceptability	None	3	
Feasibility	None	3	
Desirability	Minor	3	“use space and type size more effectively” “the voice and tense should be the same for all” “The colour yellow used in the original version … can impede performance and use of some types of writing instruments pencils and ball point pens.” “The red fraction should be at the top of the figure” Correct alignment to “support a professional and credible look to your document and therefore detracts from its content.” Revise version date to be less obvious (so over time does not look outdated). Move funding logos to the back. Change visual analog line for values to response bubbles. Revised back panel to provide a more cohesive flow to the pamphlet allowing the surgical risks to be viewed more readily and placing the funding source on this page.

On the basis of the results of Cycle 3, the color scheme was changed, the alignment of the tool was revised and the values exercise was changed from a visual analog scale to discrete response options. The final version of the decision aid prototype is shown in Fig. 2.

## Discussion

This paper outlined the process of developing a decision aid prototype for parents considering adenotonsillectomy for their children with sleep disordered breathing. Parents indicated that they wanted a reliable resource that provided them with information on the symptoms of sleep disordered breathing, management options, and the risks and benefits for each of these management options [18]. Using an iterative approach, we collected feedback on the comprehensibility, acceptability, and feasibility of the decision aid prototype from parents, nurses, and otolaryngologists. We also obtained informal feedback from decision aid researchers and design experts. The stakeholders informed the development of the decision aid prototype to support shared decision-making during surgical consultation.

Decision aids are one method of promoting shared decision-making [24], but to date, there are no widely used decision aids in pediatric otolaryngology [9]. The current study aimed to address this need by designing a decision aid specific for parents of children with sleep disordered breathing, which is a very common condition in pediatrics. Although there are different approaches to the development of a decision aid, we used the best practices recommended by the IPDAS Collaboration. The IPDAS recommends an iterative process, which allows multiple stakeholders (e.g. parents and healthcare providers) to define their needs so that the decision aid will be feasible and useful to all stakeholders. Although there are a number of decision aids available, many have been created without following a specific methodology, resulting in an increased chance that they may not have adequate reference sources or may be biased in how the information is presented [25]. Establishing a structured method for development of a decision aid includes methods to review the evidence regularly and update details to stay current with evidence and stakeholders’ expressed needs.

A very recent article described the development process of a decision aid for adult patients with obstructive sleep apnea [26]. Although a similar methodology was used in developing the decision aid, the content, including the treatment options, was different than the current decision aid for pediatric patients as the two populations have very different causes of sleep disordered breathing. As well, compared to adult medicine, there are often more stakeholders involved in the decision-making process in pediatrics (e.g, both parents). Therefore, it is necessary to have two separate tools addressing sleep disordered breathing in pediatrics and in adults, as the requirements for decision-making in the two populations are unique [27].

Shared decision-making is considered to be a critical component of best practice in medicine [2]. Shared decision-making has particular utility for treatment decisions which do not always have a single superior option because it incorporates patient values to make an optimal decision for the individual patient [24]. Shared decision-making has the potential to improve patient knowledge and outcomes, decrease decisional conflict and variations in unnecessary medical practice, improve adherence to practice guidelines, and ease costs to the healthcare system by reducing the number of patients receiving unnecessary treatments [24, 28]. Specialties such as otolaryngology are optimal settings for shared decision-making since many treatment options are considered elective and some of these options have mixed evidence [9, 28].

The IPDAS methodology has been used to develop decision aids that can be applied in a variety of medical situations including reconstructive surgery following mastectomy [29], management of heart failure [30] and management of renal failure [31]. In studies that did not utilize the IPDAS framework, the quality of the decision aid produced has been questioned [32]. Using a structured development process like the IPDAS framework ensures that the comprehensibility, acceptability, and feasibility of the tool meet the needs of the individuals the decision aid was intended for.

In this study, both parents and healthcare providers felt that with some changes to the initial decision aid prototype, the tool would be feasible to implement into clinical practice and would allow parents to become more involved in shared decision-making by providing them with information on the choice to be made, and helping them to clarify their personal values on the decision. By gathering feedback on the comprehensibility, feasibility and aesthetics of the prototype, we have aimed to create a tool that will be useful to stakeholders. This in turn will help to increase uptake of the tool once it is in its final form.

Limitations of this study are common to the literature of decision aid development. Feedback on our decision aid prototype was obtained from only two healthcare centers (one in Eastern Canada and one in Western United States), which is a potential threat to the generalizability of the tool [29, 30]. We plan to obtain more feedback from providers at other centers to help mitigate this issue. However, it is to be noted that data saturation occurred with the iterative qualitative process, which indicates that further feedback may not have necessarily generated new data. As well, several other decision aid prototype development studies used similar number of stakeholders [29–31, 33, 34]. Clearly, a pilot trial is necessary to assess for wider usability, which will be followed by a randomized controlled trial to assess the effectiveness of the decision aid before the tool can be implemented into routine clinical practice.

Another related limitation is that we did not consider the cultural variations that may exist in other healthcare centers (i.e., our population was fairly homogenous [19]). Future testing of cultural sensitivity of the tool, therefore, may be beneficial in the wider application of the decision aid outside of our center [30].

The timing of when this tool should be available to parents and how this may relate to the slowing of consultation time (if presented during the consultation) was another area of feedback where we had variations in response. Timing of when a decision aid is made available can impact the effectiveness of the tool [29]. Therefore, we will address this issue in the next phase during a multicenter pilot trial by introducing the decision aid tool at different times (pre-consultation/during/post-consultation) to ensure that the length and quality of the consultation remains adequate. In turn, the uptake of the decision aid into clinical practice will be optimized.

In addition to the differences among parents regarding when they may find the decision aid useful, individual providers may vary in how they use the tool. Individual surgeon differences in values may bias how or when the decision aid is used. Some providers may have long-standing practice patterns that already incorporate shared decision-making and may elect not to use the decision aid. Also, slightly different complication rates may exist among providers and institutions, so adapting figures and data to each provider or healthcare center may be beneficial.

Finally, the decision aid may not be applicable to all children presenting with sleep disordered breathing. For instance, children with severe obstructive sleep apnea documented on a sleep study may not improve over time without surgical intervention [35]. Therefore, the decision to proceed with surgery could be considered less challenging and a decision support tool may not be necessary since benefits can be deemed to clearly outweigh the risks. On the other hand, some children (e.g., those with mixed apneas, craniofacial anomalies) may require other interventions that are not listed in the decision aid [e.g., continuous positive airway pressure therapy (CPAP)] to improve their sleep disordered breathing. We decided not to include multiple therapies (e.g., CPAP, nasal steroid spray, orthognathic surgery) because decision aids should be simple and ideally present the most common and equivocal options. Therefore, the healthcare provider may need to discuss other therapies than what is presented in the decision aid when appropriate.

Moving forward, we will continue to follow the IPDAS methodology by conducting a multicenter randomized controlled trial using the decision aid prototype to inform further modifications, address some of the issues that may arise from differences amongst providers and institution practices, and to aid in the development of an electronic-version of the decision aid. Also, we will obtain information on how the decision aid was utilized in the clinical setting and how it should be implemented in the future. We will also conduct a quality-based assessment of our decision aid after the pilot trial using the IPDAS checklist [36]. Once we have developed a decision aid specific to pediatric obstructive sleep apnea, we hope to use the IPDAS methodology we have tailored to the pediatric setting to create other versions of the decision aid pertaining to other conditions in pediatric otolaryngology and in other areas of otolaryngology.

## Conclusion

A decision aid prototype that is systematically developed and balances the current synthesized evidence of risks and benefits, while at the same time supporting parents through the values reasoning process is a tool not previously available for parents considering adenotonsillectomy for their children with sleep disordered breathing. The current prototype was designed with the anticipation that it can be used in other healthcare centers treating children with obstructive sleep apnea due to adenotonsillar hypertrophy. Currently the decision aid prototype is paper-based, which is easy to share between clinics interested in implementing this tool. Initial qualitative results from beta-testing during the developmental process outlined in this study indicated that the key stakeholders thought the decision aid prototype as a valuable decision-making tool. However, the prototype should be tested in a pilot trial to assess how it will be utilized in actual clinical interactions, followed by a multi-center randomized controlled trial to test its effectiveness.
